# Comparative study of the role of nitric oxide in the regulation of food intake across vertebrates

**DOI:** 10.4142/jvs.25185

**Published:** 2026-06-25

**Authors:** Salaheddin Zarei, Ali Baghbanzadeh

**Affiliations:** Department of Basic Sciences, Faculty of Veterinary Medicine, University of Tehran, 14155-6453 Tehran, Iran.

**Keywords:** Nitric oxide, feeding behavior, arginine, comparative physiology, appetite regulation

## Abstract

**Importance:**

Nitric oxide (NO), synthesized from L-arginine by nitric oxide synthases (NOS), acts as a conserved signaling molecule involved in the regulation of food intake across animal taxa. Understanding its function has gained importance in veterinary medicine, animal production, and comparative physiology, especially given species-specific differences in arginine metabolism and immune-nutritional interactions.

**Observations:**

This review provides a comparative synthesis of the role of NO in appetite regulation in mammals, birds, and aquatic animals, highlighting conserved mechanisms, species-specific differences, and gaps requiring further investigation. In mammals, NO modulates hypothalamic circuits controlling satiety and hunger in a bidirectional, energy-dependent manner. Birds, lacking endogenous arginine synthesis, show reduced feed intake with elevated NO levels. In fish, NO—especially via agmatine—suppresses feeding by downregulating orexigenic signals and enhancing insulin-mediated pathways.

**Conclusions and Relevance:**

NO functions as a conserved but flexible regulator of food intake across taxa. Future studies employing molecular, nutritional, and behavioral tools are needed to clarify species-specific mechanisms and optimize NO-targeted feeding strategies in production animals.

## INTRODUCTION

The regulation of food intake is a fundamental physiological process essential for maintaining energy homeostasis, supporting growth, reproduction, immune competence, and survival in all animals [1,2]. This regulation involves a complex interplay between peripheral metabolic signals and central neural pathways, predominantly localized in the hypothalamus and brainstem [3,4]. These neural networks integrate endocrine, nutrient-derived, and environmental cues to control appetite and satiety responses, ensuring adaptive feeding behavior under varying internal and external conditions [5,6].

Among the molecular mediators involved in appetite regulation, nitric oxide (NO) has emerged as a highly versatile signaling molecule that exerts both stimulatory and inhibitory effects on feeding, depending on the physiological context and species examined. The functional role of NO in food intake regulation was first demonstrated in mammalian models, where both central and peripheral administrations of NO donors, such as L-arginine, were shown to reduce food consumption in satiated animals, acting as a negative feedback signal for satiety [7]. Conversely, inhibition of nitric oxide synthases (NOS) by pharmacological agents increases food intake, highlighting NO’s role in suppressing feeding under low motivation states [8,9,10]. These findings have since been extended to avian and aquatic species, where arginine availability and NO production influence feeding behavior, neuropeptide expression, insulin secretion, and immune function [11,12,13,14].

Although many of these regulatory pathways appear conserved across vertebrates, species-specific adaptations are evident. For instance, birds lack a functional urea cycle and cannot synthesize arginine endogenously, making dietary arginine absolutely essential for growth and metabolic regulation [15,16]. In fish, particularly teleosts, limited arginine biosynthesis restricts endogenous NO production, thereby linking dietary arginine intake more directly to physiological NO availability [17,18,19]. In invertebrates like *Aplysia*, L-arginine and NO similarly influence feeding by modulating chemosensory responses and neural excitability in a context-dependent manner [20,21].

Given NO’s conserved yet context-dependent roles in feeding regulation, a comparative analysis across mammals, birds, and aquatic species offers valuable insights into both the evolutionary conservation of appetite control mechanisms and the metabolic constraints imposed by species-specific physiology. This review synthesizes current findings on the role of NO and its precursor L-arginine in the regulation of food intake across diverse taxa, with an emphasis on shared neurochemical pathways, regulatory enzymes, and adaptive differences. Understanding these mechanisms not only deepens our comprehension of vertebrate and invertebrate feeding biology but also has potential applications in animal nutrition, aquaculture, and poultry science.

## METHODS

This review was based on a narrative literature search covering approximately 91 primary research articles and 9 reviews published between 1990–2025. Relevant articles were identified through searches in PubMed, Scopus, and Web of Science using combinations of keywords such as ‘nitric oxide,’ ‘food intake,’ ‘NOS,’ ‘L-arginine,’ ‘feeding behavior,’ ‘hypothalamus,’ and specific taxa (‘mammals,’ ‘birds,’ ‘fish,’ ‘Aplysia,’ etc.). Studies were included if they directly investigated NO’s role in feeding behavior with measurable outcomes and were published in English. Studies were excluded if they focused solely on NO’s vascular effects without feeding behavior assessment or were case reports without experimental data. Both foundational and recent studies were included to capture the historical development and current understanding of NO’s role in appetite regulation. Priority was given to primary research articles and comparative reviews addressing neurochemical mechanisms, central and peripheral NO signaling pathways, and dietary influences on arginine metabolism and feeding behavior across vertebrate species.

## OBSERVATIONS

### NO: synthesis and mechanisms of action

NO is a small, lipophilic, and highly diffusible gas that functions as a critical signaling molecule in diverse physiological processes, including vasodilation, immune response, neural transmission, and metabolic regulation [22,23]. In the context of feeding, NO plays a dual role: it can either inhibit or stimulate food intake, depending on the site of action, concentration, species, and physiological state [24].

NO synthesized from L-arginine via the activity of NOS, which exist in three main isoforms: neuronal NOS (nNOS), endothelial NOS (eNOS), and inducible NOS (iNOS) [22,23]. These isoforms differ in their tissue distribution, calcium dependence, and regulatory mechanisms [25,26].

**Fig. 1:**
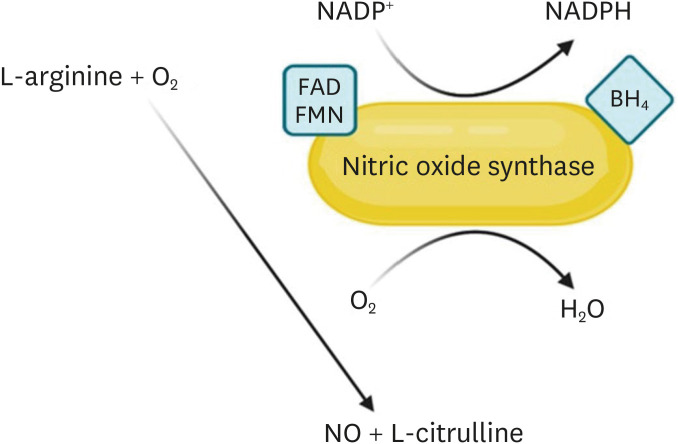
Synthesis of NO and related products. Modified based on [29]. NADPH, reduced nicotinamide adenine dinucleotide phosphate; FAD, flavin adenine dinucleotide; FMN, flavin mononucleotide; BH_4_, tetrahydrobiopterin; O_2_, oxygen; H_2_O, water.

These isoforms catalyze the conversion of L-arginine into NO and L-citrulline, utilizing oxygen and NADPH, and requiring cofactors such as FAD, FMN, tetrahydrobiopterin (BH_4_), and calmodulin [23,27,28,29]. NO synthesis catalyzed by NOS is shown in Fig. 1.

The constitutive isoforms, nNOS and eNOS, are regulated by intracellular calcium levels and produce basal levels of NO for physiological signaling. In contrast, iNOS is expressed in response to pro-inflammatory cytokines such as tumor necrosis factor-α and interleukin-1β, and generates large amounts of NO independent of calcium, particularly during immune activation [25,30,31,32,33].

The activity of NOS is closely linked to the availability of its substrate, L-arginine. This dependency becomes especially relevant in cells where arginase competes with NOS for arginine, potentially reducing NO synthesis and shifting metabolism toward urea and ornithine production [26,34]. Elevated arginase activity or low arginine availability can result in NOS uncoupling, leading to the production of superoxide radicals instead of NO, contributing to oxidative stress and impaired cellular function [35,36]. Carbamoyl phosphate synthase (CPS) catalyzes the formation of carbamoyl phosphate from ammonia and bicarbonate in the mitochondria, initiating the urea cycle and providing a precursor for arginine synthesis [37,38]. This step is crucial for nitrogen disposal and arginine biosynthesis via the ornithine cycle [28].

In mammals, NO acts through both cyclic GMP (cGMP)-dependent and cGMP-independent pathways. The primary mechanism involves activation of soluble guanylate cyclase (sGC), which catalyzes the formation of cGMP from GTP. cGMP serves as a second messenger to regulate ion channels, neurotransmitter release, and gene expression [39,40,41]. This pathway is critical for NO-mediated effects on feeding, especially in hypothalamic nuclei such as the arcuate nucleus (ARC), paraventricular nucleus (PVN), and ventromedial hypothalamus (VMH), which are central to appetite control [7,24].

Evolutionarily, NO signaling pathways are highly conserved. Bacterial NO synthases share structural similarities with mammalian enzymes, and genes encoding NOS isoforms have been identified in a wide range of organisms, including invertebrates, mollusks, and fish [42,43,44,45]. Interestingly, in *Aplysia*, NO is produced in neurons involved in feeding behavior, such as the C2 neuron, where it facilitates arousal and food-seeking behavior [46]. At the same time, it can exert inhibitory effects in response to sustained odor exposure, underscoring its complex and context-specific regulatory roles [47,48].

In birds and fish, the synthesis and function of NO are closely tied to dietary arginine availability, due to their limited endogenous arginine biosynthesis. Birds lack a functional urea cycle due to the absence of key enzymes like CPS I, making them completely dependent on dietary arginine for NO production [15,16,17,49]. Similarly, many teleost fish exhibit low activity of enzymes like CPS III and Ornithine transcarbamylase (OTC), further constraining arginine-based NO synthesis [18,50,51]. As a result, arginine supplementation in these species has been shown to enhance NO production, influencing vascular tone, immune response, and feeding behavior [19,52].

Although the fundamental enzymatic pathways of NO synthesis are conserved, notable interspecies differences in arginine metabolism, nitrogen excretion, and enzyme activity shape how NO signaling operates in different taxa. A comparative summary of these metabolic and physiological differences is provided in Table 1.

**Table 1:** Comparative overview of NO synthesis and arginine metabolism across species

Feature	Mammals	Birds	Fish
Arginine synthesis capacity	Endogenous (semi-essential)	Absent (fully essential)	Limited (species-dependent)
Main nitrogen excretion type	Ureotelic	Uricotelic	Ammonotelic/ureotelic
Key limiting enzymes	-	CPS I, OTC absent	Low CPS III activity
NOS isoforms expressed	nNOS, eNOS, iNOS	nNOS, eNOS, iNOS	nNOS, eNOS, iNOS
Dietary arginine sensitivity	Moderate	High	High
Arginase competition for arginine	Present	Present	Present
Known NO-linked feeding effects	Bidirectional	Primarily anorexigenic	Primarily anorexigenic (e.g., agmatine)

NO, nitric oxide; CPS I, carbamoyl phosphate synthase I; OTC, ornithine transcarbamylase; CPS III, carbamoyl phosphate synthase III; NOS, nitric oxide synthases; nNOS, neuronal nitric oxide synthases; eNOS, endothelial nitric oxide synthases; iNOS, inducible nitric oxide synthases.

### Role of NO in the regulation of food intake across species

#### Role in aquatic animals

In aquatic vertebrates, particularly teleost fish, NO plays an important and highly context-dependent role in feeding regulation. The metabolic conversion of proline to arginine via pyrroline-5-carboxylic acid has not been established in fish [53].

Arginine is metabolized via NOS isoforms—nNOS, eNOS, and iNOS—which are all expressed in fish tissues, including the brain and gastrointestinal tract [19]. Basal NO production by nNOS and eNOS supports functions such as vascular tone regulation and neurotransmission, while iNOS activity increases markedly during inflammation or in response to bacterial challenges [54,55].

The relationship between NO and food intake in fish has been explored through dietary interventions and intracerebroventricular (ICV) administration of arginine derivatives. One of the most notable findings is the anorexigenic effect of agmatine, a biogenic amine derived from arginine decarboxylation. When agmatine is injected into the brain, it causes a rapid and sustained reduction in food intake, with effects lasting up to 8 h [56,57]. This suppression of feeding is associated with a downregulation of orexigenic neuropeptides, such as neuropeptide Y (NPY) and agouti-related peptide (AgRP), and an upregulation of anorexigenic genes including melanocortin 4 receptor (MC4R) and insulin receptor substrate 2 (IRS2) [12,58,59].

Further, agmatine-induced anorexia correlates with transient increases in insulin secretion, suggesting that peripheral metabolic signals also play a role in NO-related feeding regulation in fish [57]. This aligns with the known role of insulin in appetite suppression, as central insulin administration reduces feeding in both fish and mammals [60,61,62].

Interestingly, while NO often suppresses feeding in teleosts, responses are species-specific and dose-dependent. For example, studies in *Scophthalmus maximus* and *Trachinotus ovatus* showed that dietary arginine elevated serum nitrite (a marker of NO production), but this did not consistently translate to enhanced growth or altered feed intake [63,64]. This suggests that while NO serves as a signaling molecule influencing appetite, its downstream effects may be modulated by additional factors, including the animal’s immune status, metabolic rate, and environmental conditions.

Beyond central mechanisms, NO also plays a role in gut–brain axis signaling in fish. Arginine supplementation has been shown to modulate NOS expression in the gastrointestinal tract and influence neuroendocrine responses associated with digestion and satiety [17,65]. Additionally, NO production in peripheral tissues contributes to vasodilation and nutrient delivery, which may indirectly affect feeding motivation and capacity, particularly under stress or hypoxic conditions [66].

In summary, the regulation of food intake by NO in aquatic animals is tightly linked to arginine metabolism, with both central and peripheral effects. While NO generally acts as an anorexigenic signal in fish—particularly when derived from agmatine or during immune responses—the effects are dynamic and species-dependent, highlighting the importance of environmental and physiological context in shaping feeding behavior.

#### Role in birds

In avian species, NO plays a multifaceted role in regulating food intake, influenced strongly by the bird’s unique metabolic characteristics and dietary requirements.

Arginine-derived NO is synthesized in birds via three isoforms of NOS (nNOS, eNOS, iNOS), as in mammals. These isoforms differ in their regulatory mechanisms and physiological functions. nNOS and eNOS are constitutively expressed and calcium-dependent, producing basal levels of NO that regulate vasodilation, gastrointestinal motility, and neural activity. iNOS is calcium-independent and is typically induced in response to inflammation, producing higher levels of NO during immune responses [25,28,67].

In the central nervous system of birds, especially within hypothalamic nuclei, NO is believed to modulate appetite through interactions with orexigenic and anorexigenic neuropeptides, although the precise mechanisms remain less defined than in mammals. Nonetheless, evidence suggests that increased NO production contributes to satiety signaling, particularly following L-arginine supplementation, which elevates plasma NO levels and reduces feed intake in broilers and ducks [68,69]. Additionally, high arginine diets have been associated with decreased hypothalamic expression of orexigenic peptides, paralleling observations in mammals [70].

Dietary manipulation studies in poultry show that elevated levels of dietary arginine—above National Research Council [71] recommendations—can influence feeding behavior, growth, and metabolic efficiency. These effects are attributed not only to NO but also to other arginine-derived metabolites such as polyamines, proline, and agmatine, which have neuromodulatory and metabolic effects [72,73]. Although agmatine’s anorexigenic role in birds has not been studied as extensively as in fish or mammals, its presence in neural tissue and its known effects in other vertebrates suggest a possible inhibitory role in feeding.

The immune-metabolic interface is another dimension where NO influences feeding behavior in birds. During infection or stress, increased iNOS activity can elevate NO levels systemically, which has been associated with anorexia and reduced growth performance [13,74]. This mirrors the inflammatory anorexia observed in mammals and supports NO’s role as a mediator of immune-induced feeding suppression.

Recent findings also suggest that dietary arginine may influence gut–brain signaling and microbiota composition, indirectly modulating feeding patterns. Arginine supplementation has been linked to improved intestinal integrity, reduced inflammation, and enhanced mucosal immunity, which are all factors known to affect appetite regulation under stress conditions [75,76,77].

In summary, the role of NO in regulating food intake in birds is intertwined with their unique nitrogen metabolism, reliance on dietary arginine, and adaptive responses to growth and immune challenges. Although fewer direct neurobehavioral studies exist compared to mammalian models, the available data support a conserved role for NO in modulating appetite, immune function, and metabolic efficiency in avian species.

### Role in mammals

In mammals, NO serves as a dynamic regulator of food intake through region-specific, state-dependent, and neurochemical interactions within the central nervous system. The hypothalamus, particularly the ARC, PVN, and VMH, represents the primary site for NO-mediated appetite modulation [7,24].

Experimental studies have demonstrated that NO exerts context-dependent effects: in satiated states, increased NO production—either through central administration of L-arginine or pharmacological NO donors—leads to suppression of feeding. This has been confirmed in rodents, where L-arginine injections into the hypothalamus reduce food intake and lower the expression of orexigenic neuropeptides such as NPY [78,79]. Conversely, inhibition of NO synthesis using NOS inhibitors, such as L-NAME, results in increased feeding, indicating that endogenous NO acts as a satiety signal [8,9,80].

During fasting or energy-deficient states, NO appears to participate in facilitating orexigenic signals. For example, blocking NO synthesis under hunger conditions impairs the expected increase in food intake, suggesting that low-level NO production may promote feeding in certain neuronal populations [81,82]. This dual role supports the hypothesis that NO modulates feeding behavior bidirectionally, enhancing intake when energy is needed and suppressing it when replete, depending on interactions with leptin, insulin, and hypothalamic neuropeptides [83,84,85].

In pathophysiological states such as obesity and inflammation, NO signaling may become dysregulated. Excessive NO, iNOS, is associated with hypothalamic inflammation, which can blunt the responsiveness to leptin and insulin, contributing to leptin resistance and overeating [24]. Furthermore, NO interacts with reactive oxygen species in the hypothalamus to form peroxynitrite, a cytotoxic compound implicated in neural damage and feeding circuit dysfunction [86,87].

Arginine metabolism plays a central role in regulating NO availability. In mammals, arginase and NOS compete for L-arginine. Elevated arginase activity can limit substrate availability for NO synthesis, leading to NOS uncoupling and generation of superoxide instead of NO [36]. Supplementation with L-arginine or citrulline can restore NO production, especially in models of metabolic syndrome or insulin resistance [88,89]. Agmatine, a decarboxylation product of arginine, also exerts regulatory effects. In rats, low doses of agmatine (10–20 nmol) have been shown to stimulate feeding and influence macronutrient preference toward carbohydrates [90,91].

Overall, NO acts as a context-sensitive modulator of mammalian feeding behavior, sensitive to both internal energy states and external interventions. Its effects are mediated through a complex neurochemical network involving NPY, AgRP, melanocortin pathways (e.g., MC4R), and interactions with systemic hormones such as insulin and leptin [61,92]. Dysregulation of NO signaling—whether through inflammation, oxidative stress, or substrate competition—has significant implications for the pathogenesis of obesity, cachexia, and metabolic disorders.

In carnivorous mammals such as dogs and cats, dietary arginine plays a critical role in NO-mediated feeding regulation due to their limited endogenous arginine synthesis capacity. The sensitivity to dietary arginine deficiency follows the ranking: cats > dogs > rats, with cats showing particularly acute responses including complete anorexia and hyperammonemia within hours [93,94,95]. This strong dependence on dietary arginine directly impacts NO production and feeding behavior in companion animals. While rodent studies have demonstrated that L-arginine supplementation reduces food intake through NO-mediated pathways [96], similar research in dogs and cats remains limited. Preliminary evidence suggests that L-arginine supplementation affects metabolic parameters in canine models, though specific research on NO’s role in satiety signaling requires further investigation. The application of comparative transcriptomic, proteomic, and metabolomic approaches to companion animal models represents a promising avenue for uncovering species-specific mechanisms of NO signaling in feeding regulation.

Across mammals, birds, and fish, experimental studies have utilized various NO modulators—including L-arginine, agmatine, and NOS inhibitors—to characterize its impact on feeding. Key findings from these studies, along with the inferred mechanisms of action, are outlined in Table 2.

**Table 2:** Effects of NO and arginine on food intake: experimental summary by taxon

Species/Model	Intervention	Observed feeding response	Mechanism inferred
Rat (satiated)	Central L-arginine	↓ Food intake	NO-mediated satiety signaling
Rat (fasted)	NOS inhibitor	↓ Food intake	NO supports hunger signals
Chicken	Dietary L-arginine ↑	↓ Feed intake	Plasma NO ↑, immune modulation
Broiler chicks	Arginine-rich diet	↓ NPY expression, ↓ feed intake	Central NO/NPY interaction
Goldfish	ICV agmatine	↓ Feed intake (rapid, sustained)	↓ NPY, ↑ MC4R, ↑ IRS2
*Aplysia*	L-arginine + food odor exposure	↓ Feeding duration	Acts as postingestion inhibitory signal

NO, nitric oxide; NOS, nitric oxide synthases; NPY, neuropeptide Y; ICV, intracerebroventricular; MC4R, melanocortin 4 receptor; IRS2, insulin receptor substrate 2.

### Comparative analysis and evolutionary perspectives

The role of NO in the regulation of food intake demonstrates both evolutionary conservation and functional divergence across mammals, birds, and aquatic animals. Despite significant differences in metabolic organization, nitrogen excretion strategies, and feeding ecology, all three groups utilize NO as a context-sensitive modulator of appetite, reflecting its deep evolutionary roots as a signaling molecule.

Across vertebrates, NO is synthesized via NOS (nNOS, eNOS, iNOS), and exerts its effects through conserved pathways, primarily by activating sGC and increasing cyclic GMP [22,23]. In the central nervous system, NO modulates hypothalamic circuits involved in appetite control—particularly those involving NPY, AgRP, and melanocortin receptors (MC4R)—a theme consistent in mammals and fish [12,24].

However, key physiological differences influence how NO regulates food intake across taxa:

• Mammals exhibit a urea-based nitrogen excretion system and can endogenously synthesize L-arginine, rendering it a conditionally essential amino acid. Their NO-mediated feeding responses are modulated by both energy status and hormonal signals such as insulin and leptin [60,83]. NO suppresses food intake during satiety and may facilitate feeding during energy-deficient states.

• Birds, in contrast, are uricotelic and completely dependent on dietary arginine due to the absence of key urea cycle enzymes [15,16]. This places tighter dietary control on NO synthesis and links feeding regulation more directly to amino acid nutrition. Despite limited direct studies, birds display a similar satiety-associated NO response and immune-related anorexia via iNOS activation [11,97,98].

• Fish show further divergence. Many teleosts have limited capacity for de novo arginine synthesis and exhibit species-specific responses to dietary arginine and NO signaling [18,19]. Agmatine, an arginine metabolite, has a particularly clear anorexigenic effect in fish, acting centrally to downregulate NPY and upregulate MC4R [56,58]. Such findings underscore both the conserved neurochemical substrates and the adaptive differences shaped by aquatic metabolism and environmental constraints.

From an evolutionary standpoint, the presence of NO-regulated feeding behavior in invertebrates like *Aplysia* supports the hypothesis that NO functioned as a primordial neuromodulator well before the divergence of vertebrate lineages. In *Aplysia*, NO participates in feeding inhibition in response to prolonged exposure to food odors, and its site-specific effects range from neural excitation to suppression depending on behavioral context [20,21]. These findings illustrate that context-sensitive NO signaling is an ancient and adaptable feature of neural systems governing feeding.

Taken together, the comparative data suggest that while NO’s biochemical synthesis and signaling mechanisms are conserved, its functional outputs have been shaped by each species’ ecological niche, metabolic constraints, and evolutionary history. Understanding these differences offers insights into how feeding behaviors are regulated across phyla and provides a framework for applying NO-targeted nutritional strategies in both basic research and applied fields like animal production and aquaculture.

## DISCUSSION

NO functions as a highly conserved but contextually flexible regulator of food intake across vertebrate species. Synthesized from L-arginine by the activity of NOS isoforms, NO participates in a complex neurochemical network integrating central, peripheral, metabolic, and immune cues that govern feeding behavior.

In mammals, NO modulates hypothalamic circuits in a bidirectional and energy-sensitive manner. Under satiated conditions, elevated NO levels—particularly from nNOS activity—suppress appetite by enhancing anorexigenic signals and dampening orexigenic pathways. In contrast, during fasting or energy deficit, low-level NO production may facilitate feeding through activation of NPY/AgRP-expressing neurons. Furthermore, during inflammation or metabolic dysregulation, excessive NO from iNOS may contribute to appetite suppression and hypothalamic dysfunction, highlighting the duality of NO’s role based on physiological state and source.

In birds, the inability to synthesize arginine endogenously due to the absence of key urea cycle enzymes links NO production directly to dietary arginine intake. Elevated NO levels—whether due to immune activation or nutritional supplementation—are generally associated with reduced feed intake. While evidence supports a central role for NO in appetite suppression, particularly through interactions with hypothalamic neuropeptides, the underlying mechanisms remain insufficiently explored. Current findings suggest that NO acts as a mediator of satiety and immune-metabolic integration, yet direct neurobehavioral studies in birds are lacking.

In fish, NO appears to function predominantly as an anorexigenic signal, particularly when derived from agmatine, an arginine metabolite with potent inhibitory effects on feeding. Agmatine reduces NPY expression, increases MC4R and insulin-related pathways, and decreases food intake in various teleost species. However, the effects of NO in fish are species-specific and modulated by dietary arginine levels, immune status, and environmental factors such as temperature and oxygen availability. The strong dependence on dietary arginine in fish, similar to birds, ties NO’s regulatory function tightly to nutritional status.

Across taxa, conserved signaling pathways such as cGMP activation and neuropeptide modulation are evident, yet physiological adaptations have shaped species-specific NO responsiveness. Mammals exhibit more nuanced, energy-state–dependent NO signaling, while birds and fish display stronger links between NO production and nutrient intake or immune status. These differences underscore the influence of nitrogen metabolism, ecological niche, and life history traits in shaping NO’s behavioral outcomes.

Interpretation of experimental results is further complicated by methodological heterogeneity. Many studies rely on pharmacological NO donors or NOS inhibitors that may produce supraphysiological effects, obscuring the natural role of NO. High doses can induce neuroinflammation or stress responses, leading to reduced feeding that may not reflect physiological signaling. Thus, future research should prioritize physiological dosing, region-specific manipulations, and the use of genetic models to dissect the true functional contributions of NO.

Despite extensive research on NO in mammalian systems, significant knowledge gaps remain regarding its precise role in the regulation of food intake across diverse species—particularly in avian and aquatic models. Existing data suggest that NO exerts context-dependent effects on appetite through conserved neurochemical pathways, yet the underlying mechanisms and interactions remain only partially understood, especially outside rodent models.

In birds, although several studies support a link between dietary L-arginine, NO production, and reduced feed intake [72,73], most research has focused on growth performance and immune modulation rather than direct neural mechanisms. The neuroanatomical localization of NOS isoforms in the avian brain, their interaction with appetite-regulating neuropeptides (e.g., NPY, CART, MC4R), and their behavioral outcomes remain underexplored. Moreover, the role of arginine-derived metabolites, such as agmatine and guanidinoacetate, in avian feeding behavior has not been systematically studied.

In aquatic animals, particularly fish, much of the available data is derived from species-specific trials with variable dietary formulations. While some studies demonstrate clear anorexigenic effects of NO and agmatine [12,56], the regulatory network integrating NO signaling with hormonal and sensory inputs—such as ghrelin, leptin, or environmental cues like temperature and salinity—remains largely undefined. Additionally, there is limited comparative data across freshwater and marine species, developmental stages, and feeding ecologies. The lack of standardized feeding assays and uniform NO quantification protocols (e.g., nitrite/nitrate plasma levels, ICV injection responses) hinders cross-species comparisons.

Another major gap lies in the molecular and -omics-level characterization of NO-regulated pathways. While a few mammalian studies have linked NO signaling to downstream targets like CREB, IRS2, or the mTOR pathway, there is a pressing need for transcriptomic, proteomic, and metabolomic profiling in birds and fish following arginine supplementation or NO modulation. Such approaches could identify tissue-specific and developmental gene expression patterns, uncover new NO-responsive genes, and clarify the interaction between NO, nutrient sensing, and immune function.

Future research should aim to

• Map the expression and activity of NOS isoforms in hypothalamic and peripheral tissues across underrepresented species.
• Investigate the interplay between NO and other appetite-related hormones, such as insulin, ghrelin, and leptin, under physiological and pathological states.
• Use genetic and pharmacological models (e.g., CRISPR/Cas9 in fish, NOS knockout lines) to dissect causality in NO-mediated feeding regulation.
• Explore the nutritional and environmental modulation of NO pathways, particularly under stressors relevant to aquaculture and poultry production (e.g., heat, hypoxia, pathogen exposure).
• Standardize methodologies for NO measurement and behavioral feeding assays to enable robust cross-study comparisons.

By addressing these gaps, future work can not only enhance our understanding of NO as a feeding regulator but also facilitate its application in precision nutrition strategies, aimed at improving health, growth efficiency, and resilience in food-producing animals.

In conclusion, NO is not merely a metabolic byproduct but a central neuromodulator in the control of food intake. Its effects are bidirectional, species-dependent, and tightly linked to the neural and metabolic architecture of each organism. Understanding these context-specific roles is essential for advancing our knowledge of vertebrate feeding regulation and for informing nutritional and management strategies in poultry, aquaculture, and veterinary sciences. The comparative analysis reveals a notable gap in direct studies of NO-mediated feeding regulation in companion animals. While the conserved nature of NO pathways across mammals suggests potential applicability of rodent findings to dogs and cats, species-specific metabolic differences warrant dedicated investigation. Future research should include transcriptomic approaches to characterize NO-related pathways in companion animal models, which could inform nutritional strategies for obesity management. Continued cross-species investigation, coupled with modern molecular and behavioral tools, will be critical for resolving outstanding questions and translating basic findings into applied outcomes.
